# COVID-19 related calcific myositis cases

**DOI:** 10.1259/bjrcr.20200120

**Published:** 2021-01-14

**Authors:** Haseeba Tawfeeq, Fiona Witham, Gurdeep S Dulay

**Affiliations:** 1 Queen Alexandra Hospital, Portsmouth NHS Foundation Trust, England

## Abstract

COVID-19 related calcific myositis is a novel entity which is not well established in the literature to date. At Portsmouth, we encountered two cases during the initial peak of the pandemic that appeared to have similar clinico-radiological features. Our cases highlight the importance of COVID-19 calcific myopathy as a potential cause of prolonged shoulder and upper limb girdle symptoms.

## Clinical presentation

### CASE 1

We present the case of a 62-year-old manual worker with a raised BMI, who presented with a 5 day history of a non-productive cough in association with progressive shortness of breath. Upon admission, the patient’s oxygen saturations were noted as 86% on 15 L/min of oxygen. A trial of CPAP (continuous positive airways pressure ventilation) did not bring about any notable clinical improvement. He was subsequently transferred to the intensive care unit for ongoing management, intubation and ventilation, including in the prone position. The COVID-19 swab test (PCR) performed on seventh April 2020 was positive.

### CASE 2

We describe a case of a 52-yearold lady with a raised BMI who was admitted to hospital with respiratory symptoms and malaise. A COVID-19 swab (PCR) sent on the first April 2020 returned as positive. She was noted to have Type 1 respiratory failure and was initially trialled on CPAP. She was admitted to the intensive care unit for invasive ventilation following clinical deterioration due to COVID-19 pneumonitis. The patient underwent a prolonged period of time on the intensive care unit (over 1 month) receiving treatment for COVID-19. She was placed in a prone position during the period of invasive ventilation on the intensive care unit.

## Investigations/Imaging findings/Treatment

### CASE 1

**Figure 1.**
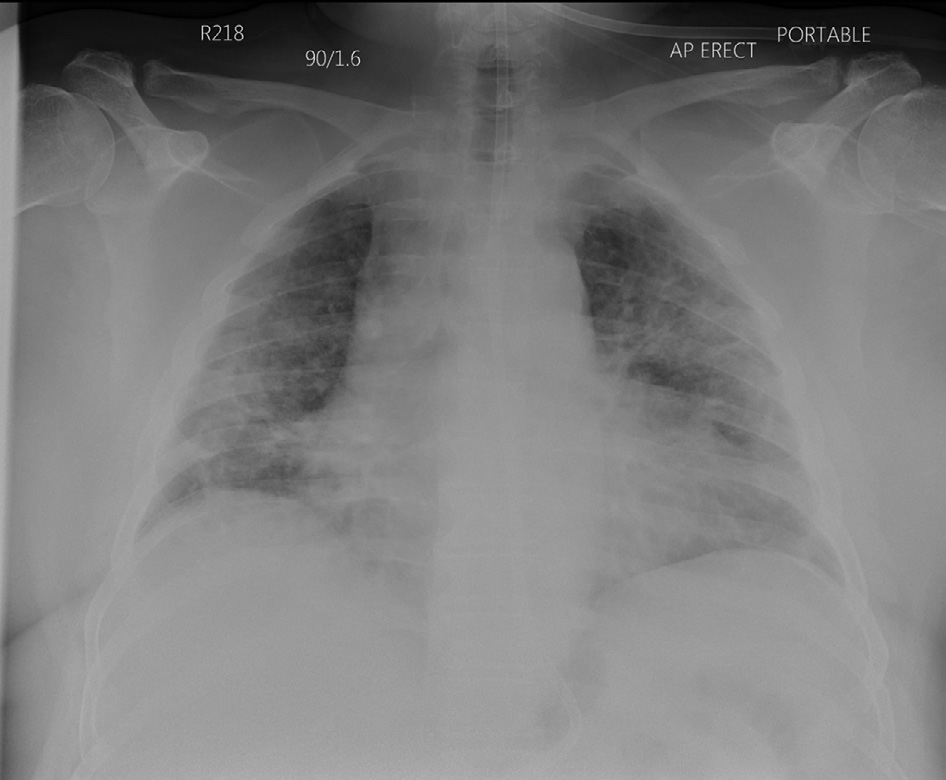
Chest x-ray (7^th^ April 2020) showing extensive bilateral airspace infiltrates, representative of COVID-19 pneumonitis.

**Figure 2.**
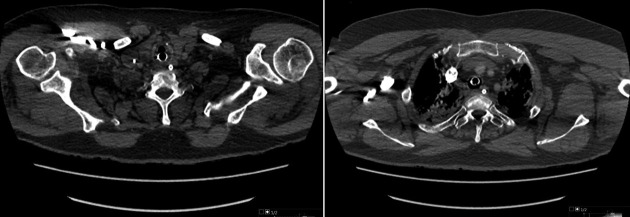
CTPA (10^th^ April 2020) performed for suspicion of pulmonary embolism and evaluation of COVID-19 pneumonia showed no measurable calcific change.

Chest X-ray (seventh April 2020) showed widespread bilateral symmetrical airspace shadowing classic for severe COVID-19 pneumonitis (see Figure 1), as did his first CTPA (10^th^ April 2020), (see Figure 2) showing a mixture of ground glass shadowing and consolidation, which was performed for increased oxygen requirement.

The COVID-19 swab test (PCR) seventh April 2020 was positive. The patient had a prolonged period of time in the intensive care unit (20 days) where he remained intubated and ventilated. As per current conventional practice, the patient was placed into a prone position whilst intubated and ventilated. This is done in order to facilitate more effective ventilation.

**Figure 3.**
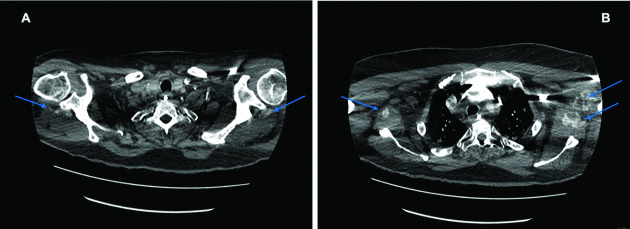
CTPA (2^nd^ May 2020) demonstrating muscle fibre long axis streaky deposits of calcification within (A) infraspinatus muscles (Arrows) and extending into (B) latissimus dorsi, subscapularis and biceps muscles bilaterally (Arrows).

The second CTPA (2 May 2020), performed for suspicion of pulmonary thrombus or embolus causing an increased oxygen requirement, showed interval improvement of the bilateral, symmetrical dense consolidations, but still moderate to severe classic COVID-19 pneumonitis and interval development of calcific myositis (see Figure 3) affecting both shoulder girdles; not seen on a prior CTPA of 10 April 2020 (see Figure 2).

**Figure 4.**
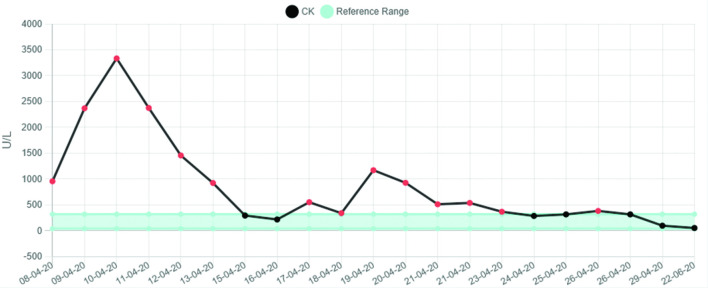
Graphical representation of CK levels.

**Figure 5.**
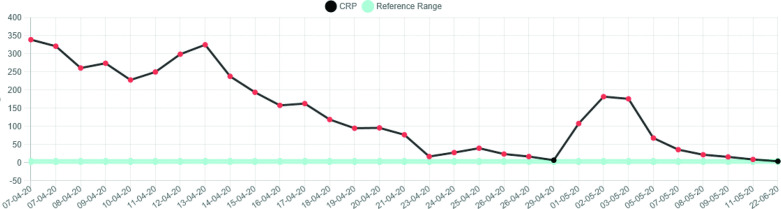
Graphical representation of CRP levels.

There was a significant rise in the CK (creatine kinase) which peaked on 10 April 2020 at 3334 U l^−1^ and normalised within a week. Subsequently, there was a further increase in the CK up to 1171 U l^−1^ before complete normalisation of this level within a further 7 days. There was associated and notable rise in the CRP (C-reactive protein) which peaked at 325 mg l^−1^. This increase in CRP level corresponded approximately to the time of elevation in the CK (see Figures 4 and 5).

Following extubation, the patient was subsequently transferred to the medical rehabilitation ward. Whilst undergoing further evaluation and rehabilitation the patient was noted to have marked pain in the proximal areas of the upper limbs and more markedly a reduction in movement at both shoulders. Physiotherapy input and analgesia resulted in some clinical improvement. He was discharged home to continue to use regular simple analgesia and continue physiotherapy exercises. However, at the time of the last review the patient reported some deterioration in shoulder symptoms, range of movement at both shoulders and reported muscular pain in the proximal upper limbs. The patient remains otherwise systemically stable.

### CASE 2

**Figure 6.**
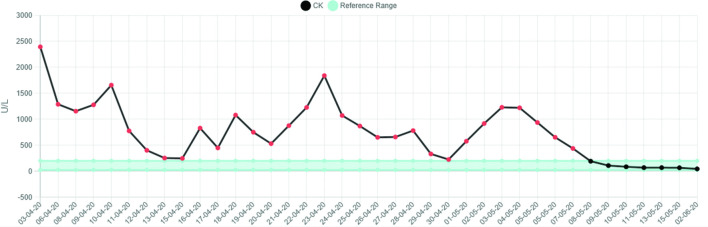
Graphical representation of CK levels.

**Figure 7.**
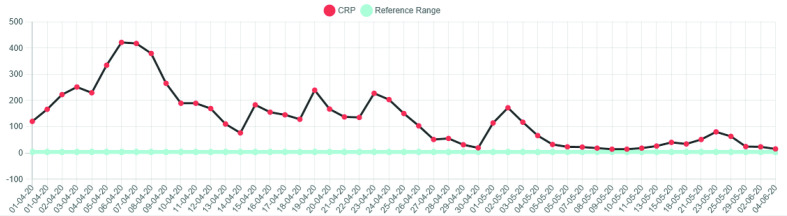
Graphical representation of CRP levels.

**Figure 8.**
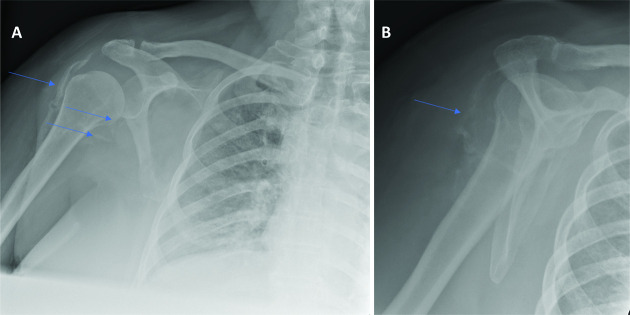
Right shoulder X-rays (1^st^ June 2020). (A) AP view showing sheet-like calcification within the lateral aspect of deltoid with streaky deposits of calcification within the subscapularis and latissiumus dorsi musculotendinous junctions (Arrows). (B) Lateral scapular view showing sheet like calcifications within the posterior deltoid.

**Figure 9.**
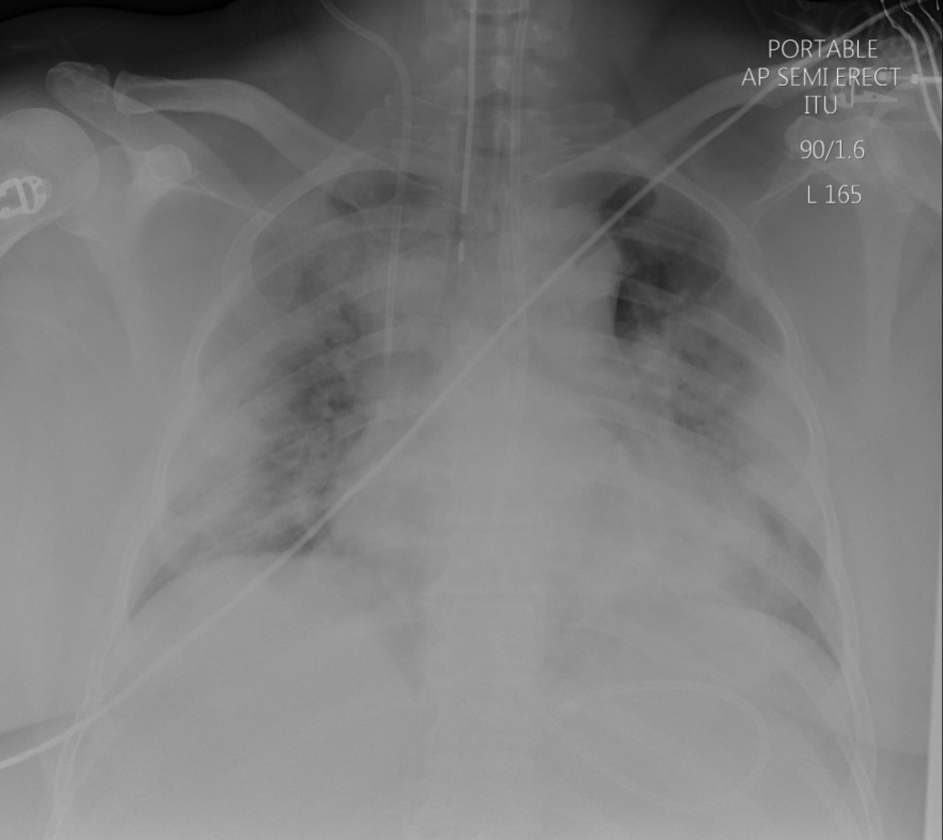
chest X-rays of 1 April 2020 obtained to evaluate covid-19 pneumonia status. No calcifications seen about the part imaged shoulders.

Similarly to the first case, there was an observed increase in the CK. The peak CK was 2394 U l^−1^ on third April 2020. There was an associated systemic inflammatory response, the CRP level reaching a maximum level of 421 mg l^−1^ 3 days after the peak CK level (see Figures 6 and 7). After successful extubation and initial close observation, the patient was transferred to a medical ward for ongoing rehabilitation. During the phase of early mobilisation, the patient had a number of symptoms particularly with pain and a restricted range of movement in both shoulders. Subsequent radiographs of first June 2020 demonstrated bilateral calcific myopathy, more severe on the right (see Figure 8). This was an interval development since a prior chest radiograph was obtained on first April 2020 which showed no calcific change on the part imaged shoulders (see Figure 9).

## Discussion

Our two cases appear to have similar clinico-radiological features. Firstly, both patients had a prolonged period of time in intensive care and were placed into the prone position for the majority of that time; approximately 20 days in case 1 and 30 days in case 2. Both patients also exhibited a notable elevation in CK (both peak levels were >2000 U l^−1^) with a systemic inflammatory response (both peak CRP levels > 300). Both patients were over 50 years of age and had a raised BMI prior to admission.

Local practice through the first wave of the 2020 pandemic has been to perform COVID-19 PCR for the purpose of diagnosis and sometimes to demonstrate clearance of virus, but not to perform serial viral titres. The CRP values may initially give an indication of the viral load, but will continue to be raised through the cytokine storm, thrombotic and superadded infective complications commonly associated with severe COVID-19 disease.

The CK graph in these two patients we believe represents the activity of the acute myositis. The appearance of calcific manifestation of the myositis lagged behind the CK elevation. When the CK normalised but the calcification persisted we have termed this “calcific myopathy”, which has been accompanied by persistent symptoms. Both patients continue to have significant daily symptoms despite several months having elapsed since the initial onset of their calcific myositis. We propose that calcific myopathy may present part of the spectrum of the newly emerging phenomenon of long COVID.

Although a number of radiological features of COVID-19 disease have been described to date, musculoskeletal radiological features are neither highly prevalent nor well described in the literature to date. The musculoskeletal features of disease and mechanisms involved are not fully understood thus far. This reflects the likely rarity of the association we describe. To the best of our knowledge, these are the first two cases of COVID-19 related calcific myositis at our hospital or reported in the literature. A case of non-calcific myositis associated with COVID-19 pneumonitis has been reported in the literature.^1^

We believe it is important to be aware of the possibility of COVID-19 related calcific myositis and myopathy being a potential cause of prolonged shoulder and upper limb girdle symptoms, as this may provide important information which helps account for the symptom burden and facilitates a more tailored approach to physiotherapy and rehabilitation. Optimal or more definitive management of the COVID-19 related calcific myositis is not yet established as this is a newly emerging condition.

## Learning objectives

1. Acute calcific myositis associated with COVID-19 pneumonitis seems to be a rare phenomenon that requires clinicians’ awareness and suspicion when symptoms, signs, or biochemical features of muscle inflammation develop in the setting of COVID-19 pneumonia.
2. Symptoms persisting beyond the normalisation of CK may indicate a progression to calcific myopathy, which is thought to be part of the newly emerging phenomenon of long COVID.
3. Radiologists are reminded to be vigilant in examining the extra thoracic soft tissues on COVID-19 related chest imaging.
